# *Mycobacterium bovis* BCG Triggered MyD88 Induces miR-124 Feedback Negatively Regulates Immune Response in Alveolar Epithelial Cells

**DOI:** 10.1371/journal.pone.0092419

**Published:** 2014-04-04

**Authors:** Chunyan Ma, Yong Li, Jin Zeng, Xiaoling Wu, Xiaoming Liu, Yujiong Wang

**Affiliations:** 1 Key Laboratory of Ministry of Education for Conservation and Utilization of Special Biological Resources in the Western China, Yinchuan, Ningxia, China; 2 College of Life Science, Ningxia University, Yinchuan, Ningxia, China; Colorado State University, United States of America

## Abstract

The emerging roles of microRNAs (miRNAs) and pulmonary epithelial cells in regulating the immune response against microbial invasion has attracted increasing attention in recent years, however, the immunoregulatory roles of miRNAs in the pulmonary epithelial cells in response to mycobacterial infection has not been fully demonstrated. In this study, we show that miR-124 expression is induced upon *Mycobacterium bovis* Bacillus Calmette-Guerin (BCG) infection in A549 alveolar epithelial cells and murine lungs. miR-124 is able to modulate Toll-like receptor (TLR) signaling in A459 cells. In this regard, multiple components, including TLR6, myeloid differentiation factor 88 (MyD88), TNFR-associated factor 6 and tumor necrosis factor-α of the TLR signaling cascade are directly regulated by miR-124 in response to BCG stimulation. In addition, miR-124 expression was induced upon MyD88 overexpression and/or BCG stimulation, while silencing MyD88 expression by small interfering RNA dramatically down-regulated miR-124 transcription in A549 cells. These results indicate an underlying negative feedback mechanism between miR-124 and MyD88 in alveolar epithelial cells to prevent an excessive inflammatory response during mycobacterial infection. These observations suggest that miR-124 is a potential target for preventive and therapeutic intervention against the pulmonary tuberculosis, an infectious disease caused by *Mycobacterium tuberculosis* infection.

## Introduction

Each year, an estimated nine million people contract human tuberculosis (TB), and two million people die from the disease worldwide [1]. *Mycobacterium tuberculosis* bacillus (Mtb) is the causative agent of TB. Mtb undergoes various genomic reprogramming events upon infection, which subsequently prevent the immune system from completely eliminating latent infectious agents [2]. Despite the identification of cell-mediated immune responses and pro-inflammatory cytokines and chemokines that play crucial roles against Mtb infection, the underlying mechanism controlling Mtb adaptation remains poorly understood [3]. Mtb primarily targets the pulmonary macrophages, and thus pervious studies on immune responses against Mtb infection have focused mainly on the alveolar macrophages or dendritic cells (DCs) [4], [5]. However, increasing evidence has suggested that pulmonary epithelial cells—to which Mtb is able to directly bind and penetrate—also represent an important step in the Mtb infection process [6]–[8].

In addition to its role as a physical barrier, the pulmonary epithelium produces an array of innate immune receptors, antimicrobial proteins, pro-inflammatory cytokines and chemokines that directly target pathogens and/or recruit immune cells to the site of infection. The pulmonary epithelium is of significant importance to understanding the Mtb infection process, because pulmonary epithelial cells are both the source for initiating the host immune system, as well as the initial targets for invasion by Mtb [9]–[11]. Previous *in vitro* studies using human airway epithelial cells demonstrated that *Mycobacterium bovis* Bacillus Calmette-Guerin (BCG) infection activated various signaling pathways to induce the production of numerous factors important in regulating immunity including chemokines CXCL8, CXCL10, CCL5, and CCL2 [12]–[16], cytokine IL-10 [12], and antimicrobial peptides β-defensin-2, cathelicidin LL-37, and hepcidin [18]–[21]. Moreover, Chuquimia et al. recently showed that airway epithelial cells were able to internalize and restrict BCG growth, functioned as antigen presenting cells for primed T cells [13], and produced a secretion profile that differed from that seen from pulmonary macrophages [14].

MicroRNAs (miRNAs, or miRs) are small non-coding RNA molecules, which predominantly function to negatively regulate gene expression by complementing to the 3′ untranslated regions (3′UTRs) of messenger RNAs (mRNA) [15]. Specifically, miRNAs have been shown to regulate the inflammatory response during immune cell lineage commitment, differentiation, and maturation [16]. Furthermore, miRNAs regulate the macrophage immune response and the Toll-like Receptor (TLR) signaling pathway [17]. For instance, miR-223 was reported to negatively regulate both the proliferation and activation of neutrophils by targeting myeloid Elf1-like factor 2C [18]; miR-125b and let-7 were down-regulated in response to lipopolysaccharide (LPS) stimulation in macrophages, in which the miR-125b regulated the immune response by targeting tumor necrosis factor (TNF)-α mRNA [19], whereas let-7 through a mechanism of targeting IL-6 mRNA [20]; miR-21 has also been found to be able to further negatively regulate LPS-activated TLR4 signaling by targeting the tumor suppressor gene, Programmed Cell Death 4 (PDCD4), which in turn decreased nuclear factor kappa-light-chain-enhancer of activated B cells (NF-κB) activation and resulted in the production of anti-inflammatory cytokine IL-10 [21]. Similarly, miR-146a has been demonstrated an ability to inhibit TLR signaling, subsequently inhibit the production of inflammatory mediators, by targeting TNFR-associated factor 6 (TRAF6) and IL-1R-associated kinase 1 (IRAK-1) [22]. However, the role of miRNAs in the regulation of immune response in pulmonary epithelial cells in response to microbial infection has not been established.

miR-124 is a miRNA enriched in the brain and plays a crucial role in gastrulation and neural development [23], [24]. In addition, the expression of miR-124 was suppressed in several types of cancer, including medulloblastoma, glioma, oral squamous cell carcinomas and hepatocellular carcinoma [25]–[27]. Interestingly, its expression was down-regulated in breast cancer specimens and cell lines, and enforced expression of miR-124 showed a reduction in cell motility and invasion in human breast cancer cells [28]. Over expression of miR-124 was linked to the suppression of inhibitory member of apoptosis-stimulating protein of p53 (iASPP), which subsequently led to an increased expression of NF-κB (p65) [29], [30]. Treating patients suffering from immunosuppressed glioblastoma with the systemic administration of miR-124 or adoptive miR-124–transfected T-cell transfers resulted in a remarked up-regulation of IL-2, interferon (IFN)-γ, and TNF-α [31]. These studies imply that miR-124 may performs an immunoregulatory role in epithelial cells.

In light of the above findings, we hypothesize that miR-124 performs a role in the regulation of the immune response in pulmonary epithelial cells during mycobacterial infection. To this end, we interrogated the function of miR-124 in an in vitro infection system using a human alveolar epithelial cell line, A549 cells, and in vivo murine lungs, with *Mycobacterium bovis* Bacillus Calmette-Guerin (BCG). We found that miR-124 was induced upon BCG stimulation, which in turn attenuated the inflammatory response by suppressing TLR signaling in epithelial cells. The mechanism of suppression was in part through miR-124 directly binding to multiple components of the TLR signaling pathway. Thus, this study identifies a previously unrecognized negative feedback mechanism by which miR-124 expression is able to attenuate excessive inflammation in alveolar epithelial cells in the context of mycobacterial infection.

## Results

### BCG induces miR-124 transcription in A549 cells and murine lungs

**Figure 1:**
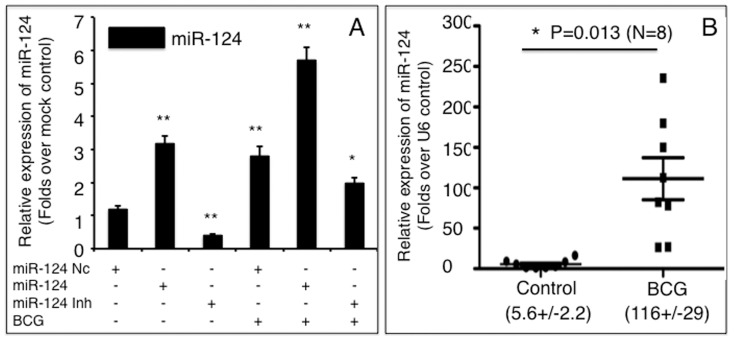
*Mycobacterium bovis* BCG induces miR-124 transcription in A549 alveolar epithelial cells and murine lungs. A: miR-124 transcript abundance in A549 cells transfected with miR-124 mimic, miR-124 inhibitor, or miR-124 nc in the presence or absence of BCG infection was determined by qRT-PCR. Fold changes in miR-124 expression are relative to untreated cells. B: The change of miR-124expression in the BCG-infected murine lungs was determined by qRT-PCR. Compared to the control group, *: p<0.05; **: p<0.01. Results represent the mean ± SD from three independent triplicated experiments (N = 9) in A, and from 8 animals in B.

In order to investigate the role of miR-124 in alveolar epithelial cells in response to mycobacterial infection, miR-124 transcripts were evaluated in A549 cells and murine lungs following BCG infection. Infection with BCG induced miR-124 expression in A549 cells by about 3-fold over non-infected control cultures. Indeed, miR-124 induction seen in BCG-infected cultures resembled cultures that had been transfected with miR-124 mimic (Figure 1). Intriguingly, an additive level of miR-124 transcription, a 5.8-fold increase in miR-124 expression, was measured when the cells were transfected with mimic and infected with BCG, in comparison with the naïve control. Additionally, the transfection of a miR-124 inhibitor decreased endogenous miR-124 by 2.5-fold and partially inhibited BCG-dependent induction of miR-124 expression (Figure 1A). Furthermore, *in vivo* study also exhibited a significant increased miR-124 transcript in the murine lung infected with BCG, in comparison with the PBS controls (p<0.05) (Figure 1B). These data suggest that miR-124 is involved in the immune response of A549 cells, as well as the murine lungs during BCG infection.

### miR-124 directly targets TLR signaling TLR6, MyD88, TRAF6 and TNF-α

**Figure 2:**
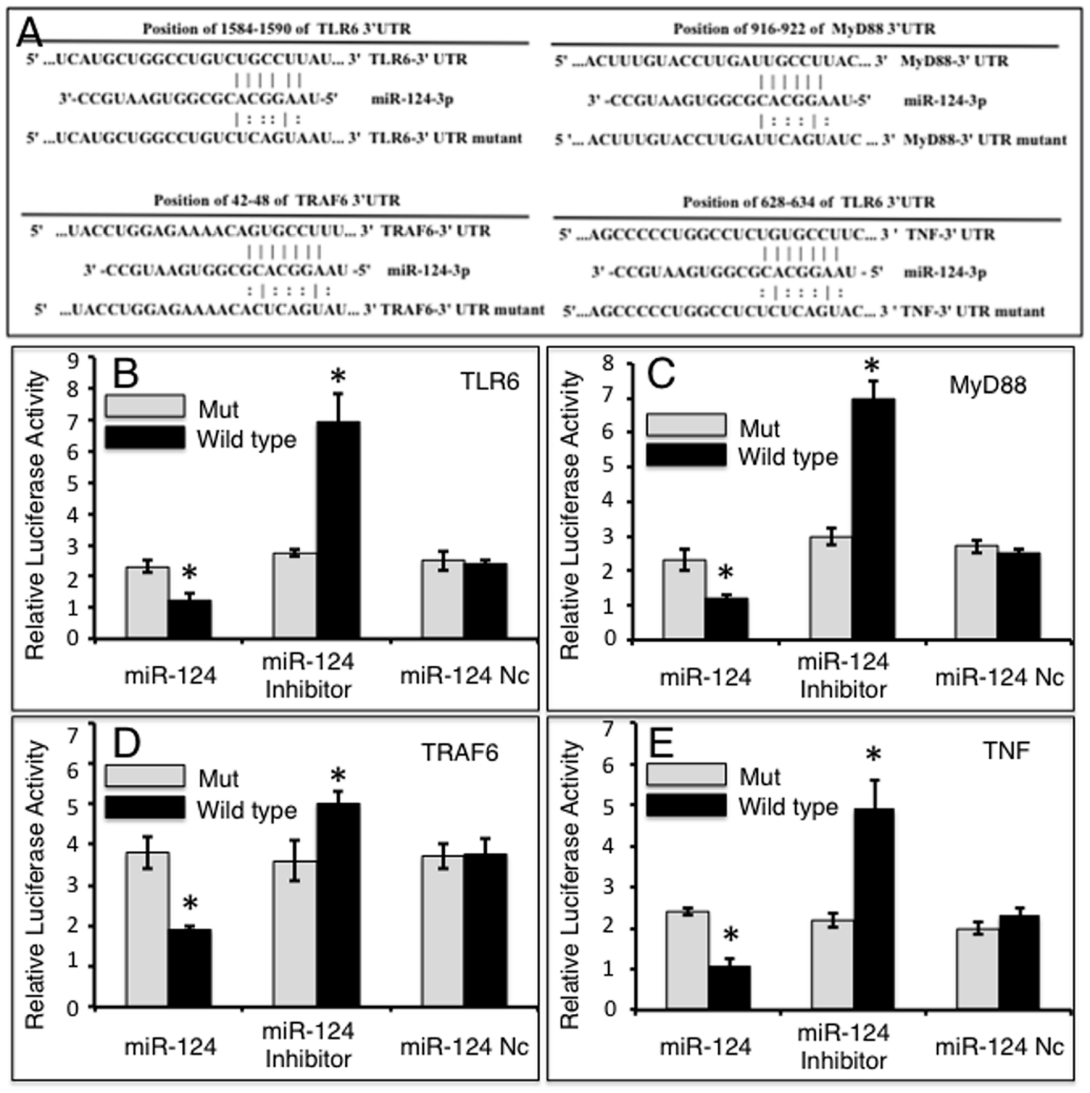
Validation of potential targets of miR-124 by a dual-luciferase reporter assay. A: Sequences of potential binding sites of miR-124 in the 3′UTR of TLR6, MyD88, TRAF6 and TNF-α mRNA (top sequences). Mutations were introduced into the putative miR-124 binding sites to generate variant 3′UTRs (bottom sequence). B-E: Dual-luciferase reporter assays were designed to validate miR-124 specificity. Cells were transfected with miR-124 mimic, miR-124 inhibitor or miR-124 control and pMIR-Report/TLR6 containing wild type or mutant 3′UTR sequence (B), pMIR-Report/MyD88 containing wild type or mutant 3′UTR sequence (C), pMIR-Report/TRAF6 containing wild type or mutant 3′UTR sequence (D) or pMIR-Report/TNF-α containing wild type or mutant 3′UTR sequence (E). Compared with pSicoR/nc group, *: p<0.05. Results represent the mean ± SD from three independent triplicated experiments (N = 9).

Since the TLR pathway is known to play a pivotal role in modulating the immune response against mycobacterial infection [32], the online computational miRNA target prediction tool, TargetScan, was used to identify potential targets of miR-124 within the TLR pathway. The TLR6, myeloid differentiation factor 88 (MyD88), TNFR-associated factor 6 (TRAF6) and TNF-α, were identified as potential targets of miR-124 by virtue of possessing several seed sequences of miR-124 within the 3′UTR of their mRNA (Table S1 in File S1). To validate whether miR-124 is able to directly target these molecules, dual-luciferase reporter vectors containing either wild-type 3′UTR sequence or the 3′UTR sequence with scrambled miR-124 seed sequences of TLR6, MyD88, TRAF6 or TNF-α mRNA were constructed (Figure 2A), and tested by co-transfecting the constructs with miR-124 mimic, inhibitor or control (Figure 2B–2E). The results showed decreased luciferase activities in cells co-transfected with miR-124 mimic and all dual-luciferase reporters harboring the wild-type 3′UTR sequences, and enhanced luciferase activities in cells transfected with miR-124 inhibitor compared to cells transfected with miR-124 control (p<0.05). Specifically, in a co-transfection experiment, miR-124 mimic reduced the luciferase activity of cells expressing pMIR-Report/TLR6 by 1.9-fold (Figure 2B), pMIR-Report/MyD88 by 2.1-fold (Figure 2C), pMIR-Report/TRAF6 by 2.0-fold (Figure 2D), and pMIR-Report/TNF-α by 2.3-fold (Figure 2E), relative to the negative control miRNA (miR-124 nc); whereas in cells co-transfected with a miR-124 inhibitor, luciferase activities were enhanced in cells expressing pMIR-Report/TLR6 by 2.5-fold (Figure 2B), pMIR-Report/MyD88 by 2.4-fold (Figure 2C), pMIR-Report/TRAF6 by 1.3-fold (Figure 2D), and pMIR-Report/TNF-α by 4.9-fold (Figure 2E) relative to miR-124 nc transfected cells. No significant change in luciferase activity was detected in cells co-transfected with either miR-124 nc or with reporters containing a mutant 3′UTR (Figure 2B–2E). Taken together, these data suggest that miR-124 regulates TLR signaling by directly binding to the 3′UTRs of TLR6, MyD88, TRAF6 and TNF-α mRNAs.

### miR-124 down-regulates MyD88 expression in A549 cells during BCG infection

**Figure 3:**
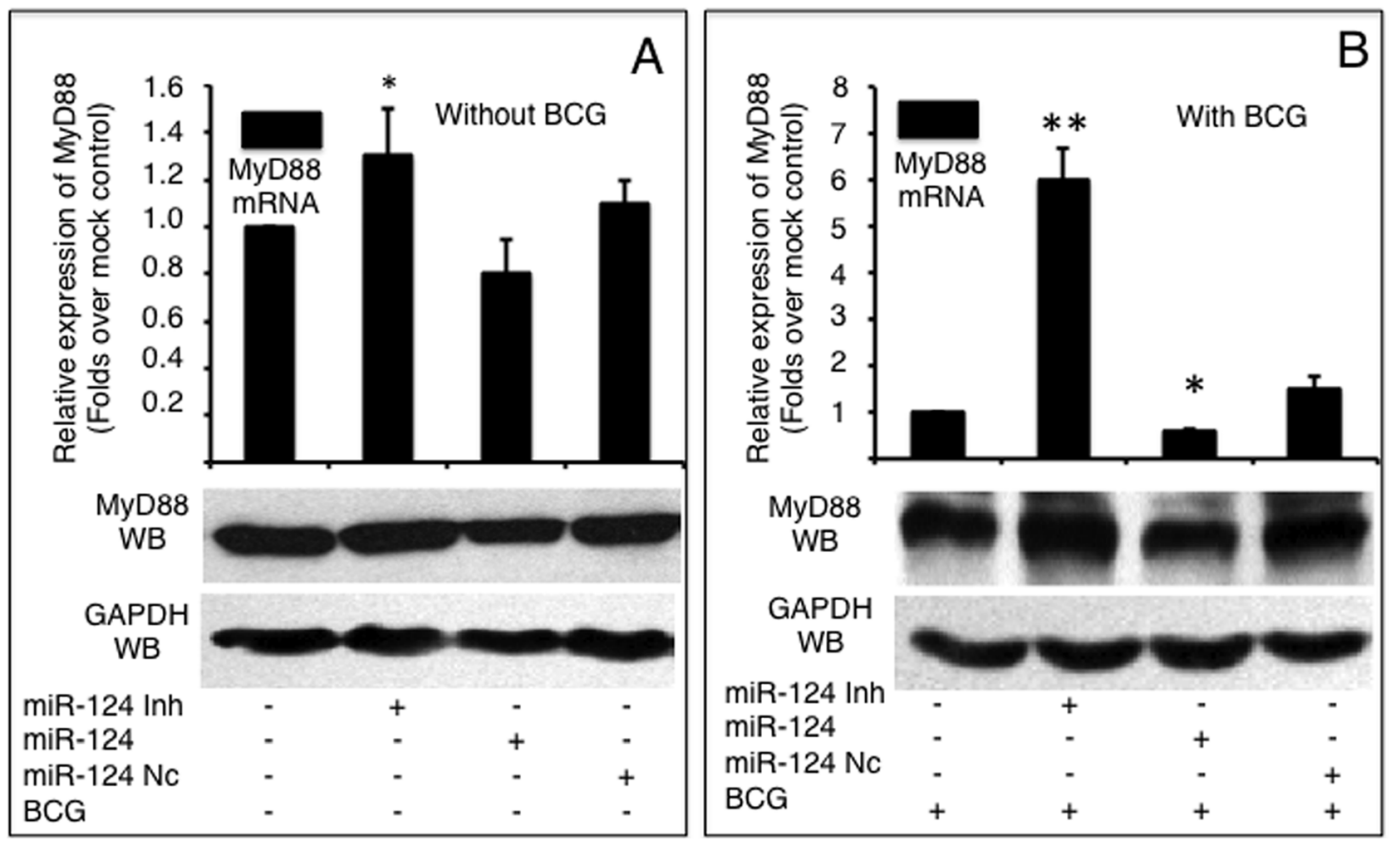
miR-124 targets MyD88 mRNA. MyD88 mRNA and protein were detected by a qRT-PCR (top panels) or an immunoblotting assay (bottom panels), respectively in naïve A549 cells (A) or cells infected with BCG (B). A549 cells were transfected with pcDNA3.1, miR-124 nc, miR-124 mimic or miR-124 inhibitor, followed by BCG infection. Comparisons were made to cells transfected with pcDNA3.1, *: p<0.05. Data represents the mean ± SD from four independent triplicated experiments (N = 12).

Because MyD88 is one of the most extensively investigated adaptor proteins of the TLR pathway, we first investigated whether miR-124 was capable of regulating MyD88 in epithelial cells during mycobacterial infection. In the absence of BCG stimulation, miR-124 mimic and miR-124 inhibitor moderately altered MyD88 abundance at both the mRNA and protein levels (Figure 3A). Importantly, following BCG infection, MyD88 expression was significantly provoked in comparison to non-infected cells (data not shown). Both MyD88 transcription and protein levels were decreased in cells introduced miR-124 mimic, and increased in cells transfected miR-124 inhibitor regardless of BCG infection; however, non-infected cells showed only a moderate alteration of MyD88 mRNA expression. A 2-fold decrease and a 1.5-fold increase of MyD88 transcript abundance were observed in BCG infected-cells that transfected with miR-124 mimic and miR-124 inhibitor, respectively (Figure 3B). Furthermore, MyD88 protein levels were reduced in BCG-stimulated cells transfected with miR-124 mimic and increased in BCG-stimulated cells transfected with pcDNA3.1 by an immunoblotting assay (Figure 3B). Taken together, these data provide evidence that miR-124 represses TLR signaling during mycobacterial infection by down-regulating MyD88.

### miR-124 represses TLR6 and TRAF6 expression in A549 cells during BCG infection

**Figure 4:**
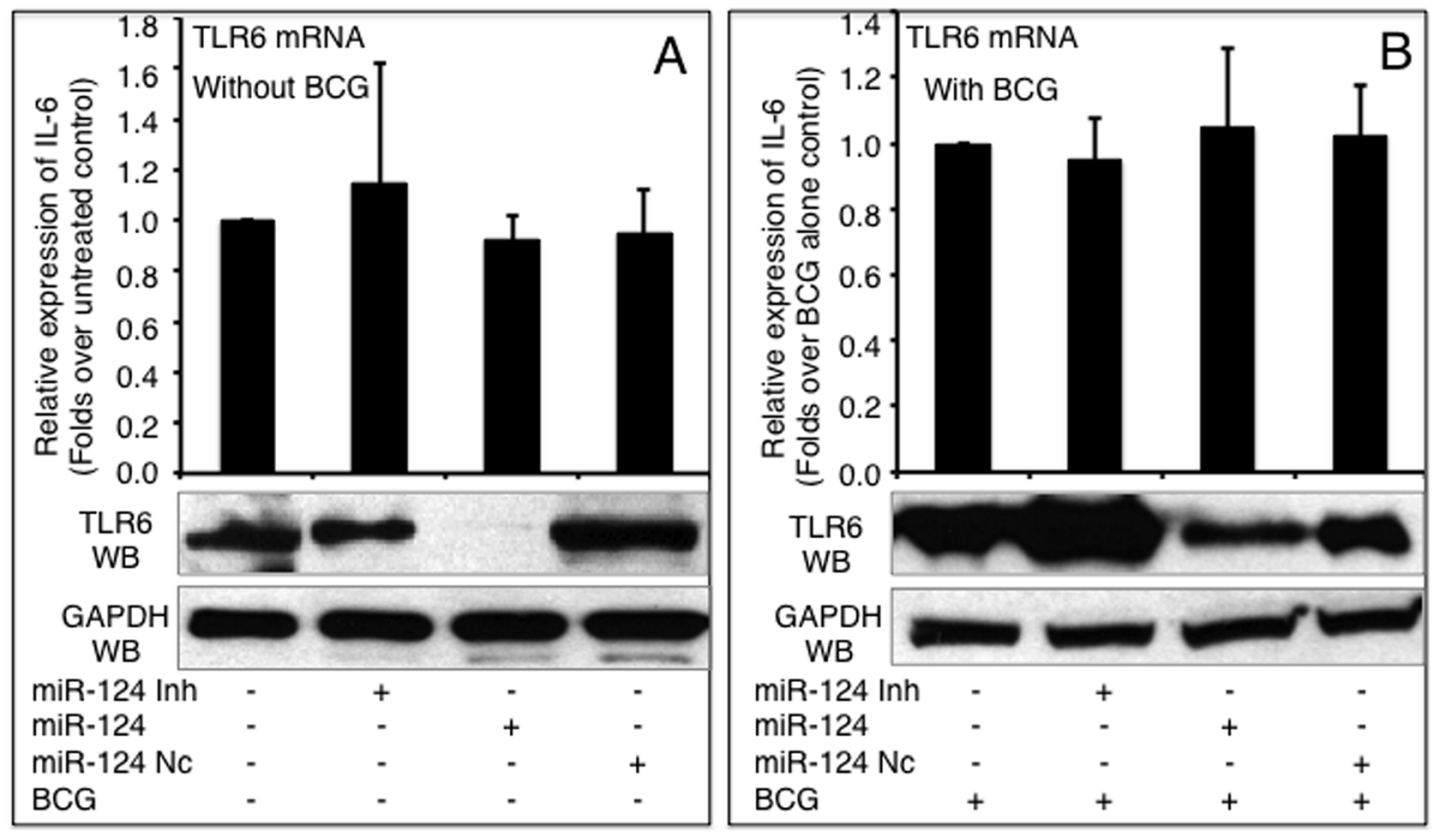
miR-124 targets TLR6 mRNA. TLR6 mRNA and protein was detected by a qRT-PCR (top panels) or an immunoblotting assay (bottom panels), respectively in naïve A549 cells (A) or cells infected with BCG (B). A549 cells were transfected with pcDNA3.1, miR-124 nc, miR-124 mimic or miR-124 inhibitor, followed by BCG infection. Comparisons were made to cells transfected with pcDNA3.1, *: p<0.05. Data represented the mean ± SD from three independent triplicated experiments (N = 9).

**Figure 5:**
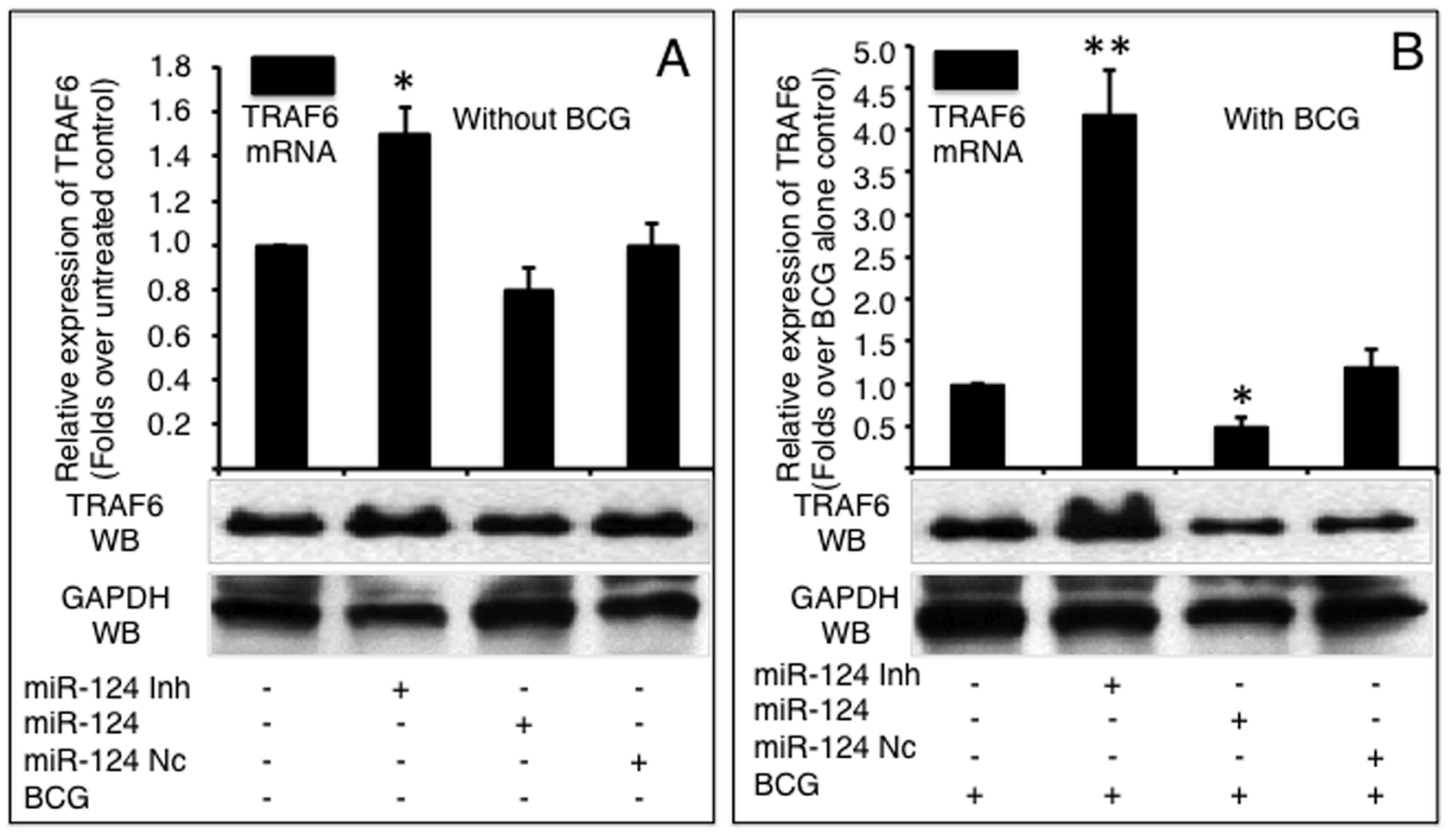
miR-124 targets TRAF6 mRNA. The expression of TRAF6 was detected by qRT-PCR (top panels) or immunoblotting (bottom panels) in A549 cells infected without (A) or with BCG (B). A549 cells were transfected with pcDNA3.1, miR-124 nc, miR-124 mimic or miR-124 inhibitor, followed by BCG infection. Compared with corresponding control group, *: p<0.05. Data represented the mean ± SD from three independent triplicated experiments (N = 9).

The effects of miR-124 on other components of TLR signaling, TLR6 and TRAF6, were also investigated. Intriguingly, miR-124 did not alter TLR6 expression at an mRNA level; however, TLR6 protein decreased in both non-infected and BCG infected cells transfected with miR-124 mimic, despite miR-124 mimic and inhibitor only moderately altered TLR6 protein in non-infected cells (Figure 4). However, as is the case for MyD88, the introduction of miR-124 mimic and inhibitor resulted in a respectively decreased and increased expression of TRAF6 at both mRNA and protein levels regardless of BCG infection, although the non-infected cells only showed a moderate alteration of TRAF6 expression at mRNA level detected by a qRT-PCR assay (Figure 5). Of note, although the infection of BCG augmented the expression of TLR6 (Figure 4) and TRAF6 (Figure 5) at protein levels, the expression levels were dramatically repressed in BCG-infected cells that were transfected with miR-124 mimic (p<0.05). In contrast, the transfection of miR-124 inhibitor further elevated BCG-induced TLR6 and TRAF6 protein levels, in comparison with the BCG-infected control cells (Figure 4 and Figure 5). These data further indicate that miR-124 can modulate TLR signaling in alveolar epithelial cells upon mycobacterial infection.

### miR-124 mediated attenuation of the BCG-induced inflammatory response by down-regulating the production of pro-inflammatory cytokines

**Figure 6:**
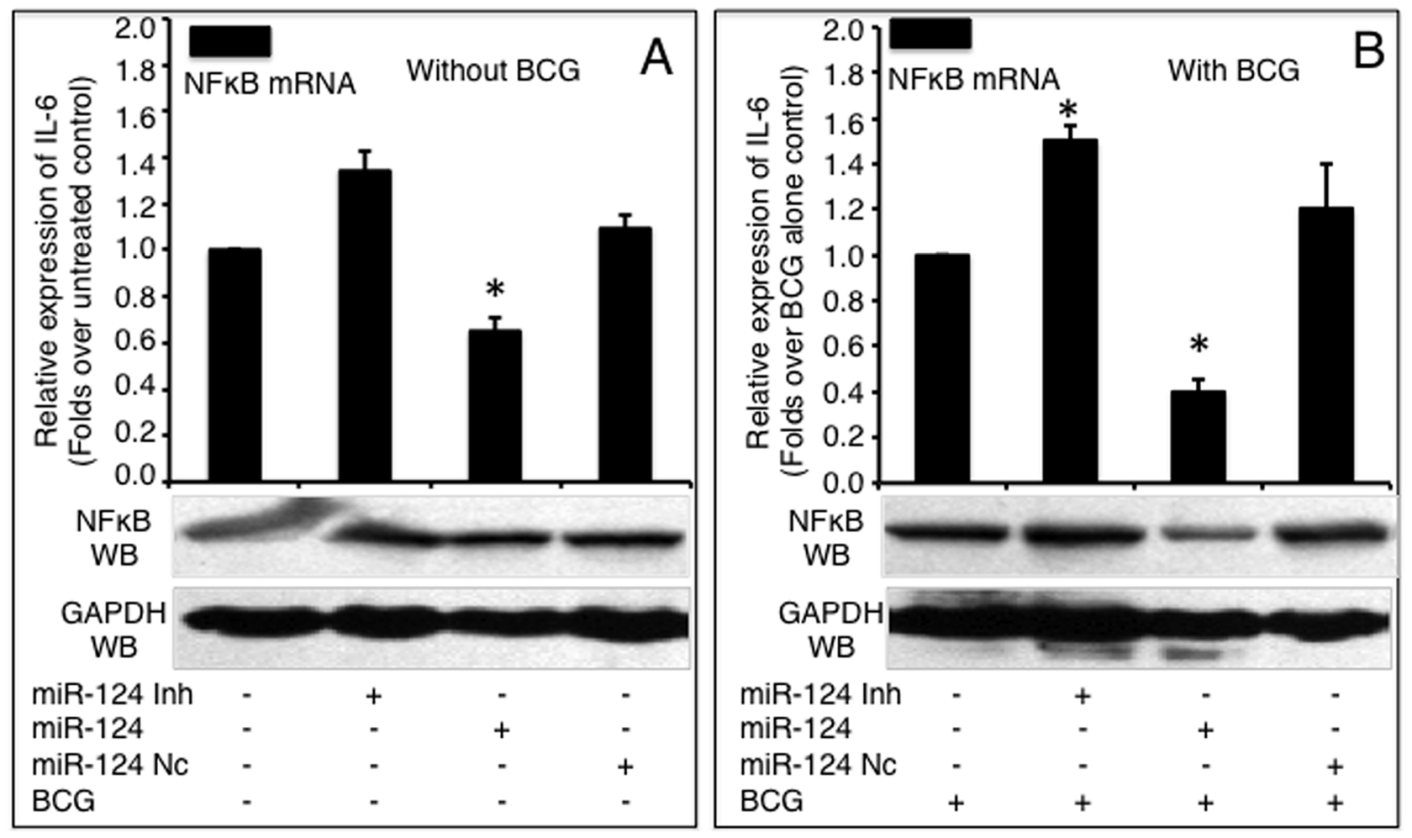
miR-124-induced down-regulation of NF-κB expression. NF-κB mRNA and protein levels were detected by a qRT-PCR (top panels) or an immunoblotting assay (bottom panels), respectively in naive A549 cells (A) or BCG-infected A549 cells (B). A549 cells were transfected with pcDNA3.1, miR-124 nc, miR-124 mimic or miR-124 inhibitor, followed by BCG infection. Compared with pcDNA3.1 group, *: p<0.05. Data represented the mean ± SD from three independent triplicated experiments (N = 9).

**Figure 7:**
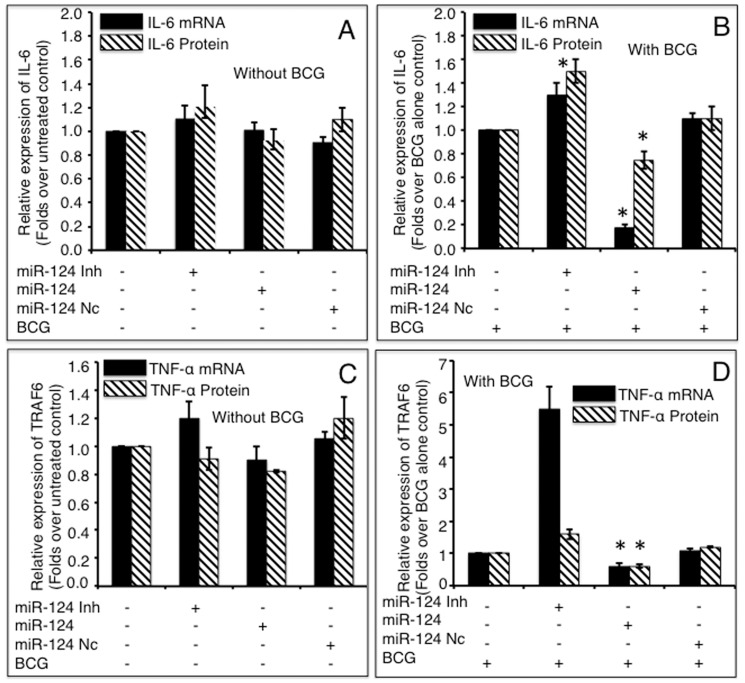
Impacts of miR-124 on the expressions of TNF-α and IL-6. A549 cells were transfected with pcDNA3.1, miR-124 mimics, miR-124 inhibitors or miR-124 nc, followed by BCG infection. The total RNA and cell culture medium were harvested at 24 h post infection. The expressions of IL-6 (A, B) and TNF-α (C, D) were determined by assays of a qRT-PCR (mRNA, solid bars) and an ELISA (protein, diagonal bars). miR-124 mimic significantly inhibited IL-6 and TNF-α expressions in both non-infected (A, C) and BCG infected (B, D) cells, and inhibition of miR-124 expression significantly elevated both cytokines, compared to control groups. Compared with pcDNA3.1 control group, *: p<0.05. Data represented the mean ± SD from three independent triplicated experiments (N = 9).

To investigate the biological significance of up-regulated miR-124 upon BCG infection, we examined the transcription of pro-inflammatory target genes down-stream of TLR signaling, including NF-κB, IL-6, TNF-α, IL-1α, IL-8, IL-12α and IFN-β in A549 cells. As expected, BCG infection induced a robust immune response with increased production of NF-κB (Figure 6), IL-6 (Figure 7A–B) and TNF-α (Figure 7C–D). However, the expression of these pro-inflammatory factors decreased to different extents in cells transfected with miR-124 mimic compared to the controls (p<0.05). Both NF-κB mRNA and protein decreased in cells introduced with miR-124 mimic and decreased in cells transfected with miR-124 inhibitor, regardless of BCG infection, although the non-infected (naïve) cells showed only a moderate alteration in NF-κB expression at the mRNA level (Figure 6). In addition, the qRT-PCR results also revealed a significant down-regulation of the pro-inflammatory cytokines IFN-β (Figure S1 in File S1), IL-1α (Figure S2 in File S1), IL-8 (Figure S3 in File S1) and IL-12α (Figure S4 in File S1) in cells transfected with miR-124 mimic (p<0.05). On the other hand, the production of pro-inflammatory factors was significantly increased in cells transfected with miR-124 inhibitor, as compared with the controls (p<0.05) (Figure 6–7, Figure S1–S4 in File S1). Of interest, the transfection of miR-124 mimic or inhibitor was also moderately altered the expression of pro-inflammatory factors in BCG uninfected cells (p<0.05) (Figure 6–7, Figure S1–S4 in File S1). These data suggest that miR-124 negatively regulates MyD88 dependent-TLR signaling by directly repressing downstream gene targets of TLR signaling.

### MyD88 activates miR-124 transcription in alveolar epithelial cells

**Figure 8:**
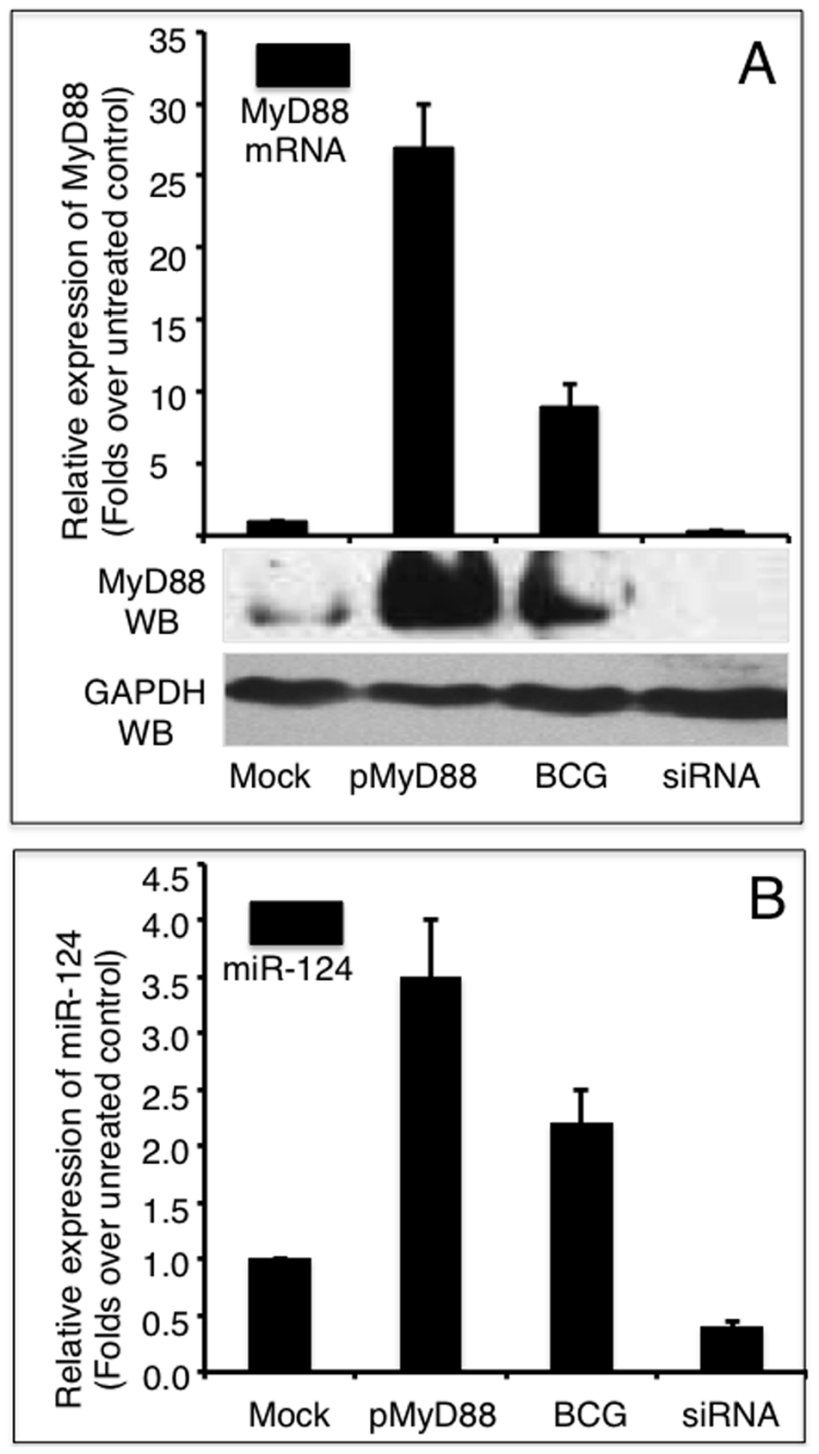
MyD88 induces miR-124 expression in A549 cells. A: A549 cells either transfected with a MyD88-expressing plasmid or infected with BCG showed markedly increased abundance of MyD88 mRNA and protein. In contrast, cells transfected with MyD88 siRNA displayed significant suppression of MyD88 mRNA and protein. B: miR-124 transcription was significantly elevated in cells overexpressing MyD88 or infected with BCG, but dramatically down-regulated when MyD88 expression was silenced by siRNA. Compared with control group, *: p<0.05; **: p<0.01. Results represented the mean ± SD from three independent triplicated experiments (N = 12).

Accumulating evidence has demonstrated that TLR signaling and cytokines induce miRNA expression [17], [33]. To gain insight into the underlying regulatory mechanism of miR-124 in the TLR pathway, we next interrogated whether MyD88 induced miR-124 transcription in A549 cells following BCG infection. MyD88 expression was altered in A549 cells by either overexpressing MyD88 from a transfected pMyD88 plasmid, inhibiting endogenous MyD88 expression by transfecting a siRNA to MyD88 (MyD88 siRNA), or by infecting with BCG. The changes in miR-124 transcripts were then assessed using qRT-PCR. Interestingly, both miR-124 and MyD88 transcripts were augmented in the cells infected with BCG (Figure 8). The transfection of pMyD88 plasmid and the BCG stimulation of A549 cells resulted in a 27-fold and 9-fold increase in MyD88 expression, respectively (Figure 8A). Substantial protein abundances were also noticeably augmented as determined by an immunoblotting assay (Figure 8B). As expected, however, the introduction of MyD88 siRNA resulted in a 70% decrease in MyD88 transcript and reduction of MyD88 protein below detectable levels (Figure 8A). Notably, the alteration in MyD88 expression was inversely correlated with the expression of miR-124. The overexpression of MyD88 was accompanied by an increase in miR-124 transcription, and the inhibition of MyD88 expression was in tandem with a decrease in miR-124 expression (Figure 8B). The miR-124 levels were increased by 3.5-fold and 2.2-fold in MyD88 overexpression and BCG stimulation, respectively (Figure 8B). Additionally, miR-124 expression was declined by 60% when endogenous MyD88 expression was repressed with siRNA. These results suggest that MyD88 is needed to induce miR-124 transcription, which in turn, attenuates the immune response in A549 alveolar epithelial cells following BCG infection. These findings suggest that MyD88 plays a critical role in mediating miR-124-dependent biological functions in alveolar epithelial cells in response to a pathogen invasion.

## Discussion

Innate and adaptive immune cells of the pulmonary mucosal surface play crucial roles in the detection and elimination of inhaled pathogens. It is now well-recognized that pulmonary epithelial cells are part of the first-line defense against infection from pathogens not only by providing a physical barrier, but also by playing indispensible roles in the initiation, regulation, and resolution of innate and adaptive immune, through mechanisms by expressing a broad array of immune response genes that compose a major part of the innate and adaptive immune response [8], [11]. However, an excessive immune response may lead to acute or chronic inflammatory disorders. Host organisms thus employ various layers of negative regulation to maintain immunologic homeostasis. In this study, we investigated the function of miR-124 in A549 alveolar epithelial cells in response to BCG infection. Among the predicted targets of miR-124, we first experimentally confirmed interactions between miR-124 and TLR6, MyD88, TRAF6 and TNF-α, and showed that miR-124 significantly attenuates the immune response upon BCG stimulation by blocking components of the TLR pathway. More importantly, we found that activated MyD88 was able to induce miR-124 transcription following BCG infection. These results suggest that miR-124 plays a crucial role in moderating the immune response in alveolar epithelial cells during an infection, such as by Mtb.

TLR signaling is a crucial modulator of the immune response. The TLR pathway has important roles in detecting pathogens and in initiating an inflammatory response that subsequently primes specific adaptive immune responses during infection. Dysregulation of this process is a hallmark of inflammatory and autoimmune diseases. It is therefore important that TLR signaling is tightly regulated. Although many mechanisms for the negative regulation of TLR signals have been identified, the induction of an anti-inflammatory response and the processes by which inflammation is resolved remain incompletely understood. In this report, miR-124 was identified as a negative regulator of immune responses in alveolar epithelial cells following mycobacterial infection. miR-124 expression was induced by BCG infection and repressed TLR signaling by directly targeting multiple signaling components in the TLR pathway, including TLR6, MyD88 and TRAF6. In addition, miR-124 was able to suppress the expression of the pro-inflammatory cytokine, TNF-α. Of note, as a tumor suppressing miRNA [34], less abundant endogenous miR-124 was detected in naïve A549 cells (http://www.microrna.org), however its transcription was dramatically elevated following BCG stimulation, and an additive effect of transfection of miR-124 mimic and BCG infection on miR-124 transcript was also observed. This result indicates that the A549 cell line is a reliable *in vitro* model for investigating miR-124 function in regulating the immune response of alveolar epithelial cells in response to bacterial infection. Moreover, the introduction of miR-124 mimic or inhibitor to BCG-infected cells showed more significant alterations to pro-inflammatory cytokine expression compared to naïve cells. Intriguingly, the transfection of miR-124 mimic or inhibitor displayed moderate alterations of MyD88, TRAF6, NFκB, and several pro-inflammatory cytokine transcripts excluding TLR6, in both naïve and BCG-stimulated A549 cells. We reasoned that miR-124 was capable of targeting multiple components of the TLR signaling cascade, and targeting any of components might functionally impact on the expression of its down-stream target genes. Since the TLR signaling cascade is initiated by the activation of TLRs, the activation or repression of TLRs may alter the subsequent expression of down-stream genes [35]. As such, targeting of TLR6 by miR-124 might lead to decreased expression of down-stream targets of the TLR signaling cascade, including MyD88 and TRAF6, as well as inflammatory cytokines and chemokines, even in uninfected cells.

Given that miRNAs are capable of negatively modulating TLR signaling to maintain homeostatic inflammation, mounting evidence has also shown that components of the TLR signaling cascade are capable of altering miRNA expression in a variety of cell types, particularly in macrophages [17], [33]. For instance, TLR signals and pro-inflammatory mediators have been shown to induce miR-146a, miR-155 and miR-9 [36]–[39]. Since the same miRNAs that regulate components of the TLR pathway are induced during TLR signaling, miRNAs likely operate in positive or negative feedback loops to modulate inflammation. Indeed, the first miRNA identified to be modulated by TLR signaling, miR-146, targets TLR signaling molecules IRAK1 and TRAF6. The miR-146 expression could be rapidly induced in human acute monocytic leukemia THP-1 cells upon exposure to LPS. Such induction was NF-κB-dependent, and was further found in macrophages, bone marrow-derived monocytes, and T-cells following a stimulation of TLR ligands, including TLR-2, TLR-3 and TLR–5 [17], [22]. Another example is miR-155, one of the most extensively characterized miRNAs, could be augmented in macrophages by many types of TLR ligands through both MyD88-dependent and MyD88-independent TLR pathways [40]. miRNAs have been shown to modulate the TLR signaling pathway by targeting proteins in its signaling cascade [41], including the transcription factors [42], signaling modulators [21], and its downstream of pro-inflammatory factors [38]. These studies indicate a mutual regulation between miRNAs and TLR pathway through complex positive- and negative feedback mechanisms, by which miRNAs contribute to a fine tuned inflammatory response to pathogen invasion. In this context, infectious pathogens initially trigger TLR signaling, and miRNAs may enhance TLR signaling to assist in mounting an inflammatory reaction. However, to avoid prolonged/excessive inflammation, miRNAs eventually work to diminish pro-inflammatory signals [17].

To date, several proteins in the TLR cascade have been experimentally confirmed as direct targets of miRNAs, including MyD88, IRAK1, IRAK2, and TRAF6. The repression of these common signaling proteins leads to a broad suppression of TLR signaling. Most bacterial pathogens engage multiple TLRs to trigger several non-redundant TLR signaling cascades. Therefore, miRNA-mediated targeting of signaling proteins shared in multiple pathways serves as efficient means to attenuate robust signal activation and avoid over-amplification of a pro-inflammatory response [17]. In agreement with this notion, we found miR-124 was able to attenuate a pro-inflammatory response in alveolar epithelial cells by targeting multiple components of the TLR pathway, including the signaling adaptors of MyD88 and TRAF6, upon mycobacterial infection.

**Figure 9:**
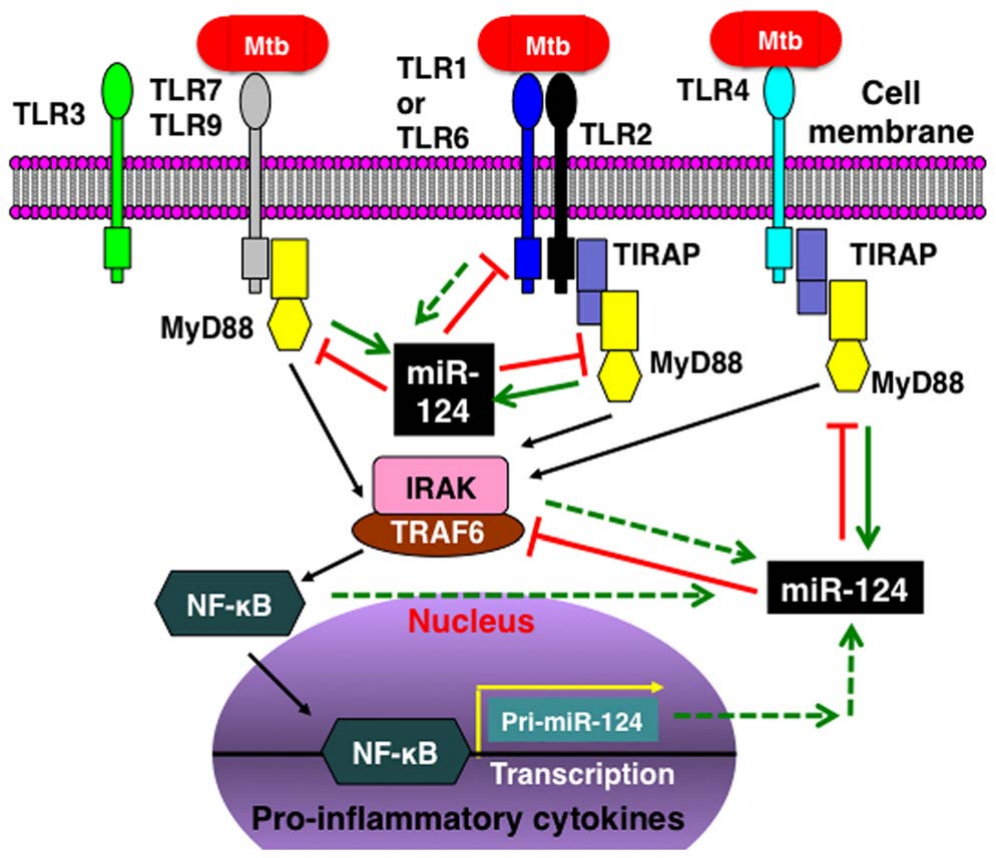
A model of the immunoregulatory role of miR-124 in alveolar epithelial cells following mycobacterial infection. Upon the invasion of mycobacteria, cellular TLRs trigger a MyD88-depentent TLR signaling cascade to initiate an inflammatory response. During the course of infection, the activated TLR signaling components and their down-stream target genes, particularly MyD88, are capable of inducing miR-124 expression, which in turn inhibits TLR signaling by directly targeting multiple components of the TLR pathway. The net result of miR-124 expression is an attenuation of the mycobacteria-triggered inflammatory response. The dashed lines indicate undefined functions.

As a critical adaptor of TLR signaling and mediator of the immune response, MyD88 initiates a cascade of signaling events eventually leading to the expression of inflammatory gene targets [43]. In an effort to identify miR-124 induction by TLR signaling, and in support of the notion that MyD88 could directly activate miR-124 transcription, we showed that MyD88 was able to induce miR-124 transcription upon BCG infection in A549 cells. Furthermore, miR-124 transcription was elevated by overexpressing MyD88 and was decreased when MyD88 expression was silenced by MyD88 siRNA. This observation demonstrated a crucial crosstalk between miR-124 and MyD88 in modulate inflammatory response, and a feedback regulatory mechanism of miR-124/MyD88 in the alveolar epithelial cells against infection, suggesting that MyD88 is a critical mediator through which miR-124 exerts its biological functions in alveolar epithelial cells in response to mycobacterial infection. In this regard, the bacterial pathogen (BCG) triggers TLR signaling activation, and augments MyD88 expression, which in turn induces miR-124 expression, subsequently the miR-124 post-transcriptionally down-regulated the MyD88 protein expression via directly targeting its mRNA and other targets in TLR signaling cascade, resulting in an attenuation of MyD88-dependent TLR signaling activity, and a substantial reduction of pro-inflammatory cytokine and/or chemokine production. As a functional consequence, the pathogen induced-pro-inflammatory response in the epithelial cells is suppressed (Figure 9). Nevertheless, additional mechanisms in TLR signaling pathway are also likely to contribute to miR-124-mediated immunoregulation in epithelial cells in response to mycobacterial infection, which needs to be further elucidated. Of note, there were several limitations existed in this study, including that the study was only performed in A549 adenocarcinoma epithelial cell line with avirulent mycobacterium BCG, but not included alveolar macrophages, and majority of the study was conducted *in vitro*. Further work will be required to fully appreciate the underlying immunoregulatory mechanism of miR-124 in alveolar epithelial cells, and the alveolar macrophages using virulent Mtb strain *in vitro* and *in vivo*.

Collectively, we identified miR-124 as a potent modulator of the immune response in alveolar epithelial cells in the present study. miR-124 exerts its function by targeting multiple components of the TLR signaling pathway, including TLR6, MyD88, TRAF6 and TNF-α. We showed that miR-124 was elevated in an alveolar epithelial cell line (A549 cells) and murine lungs upon BCG infection; the introduction of miR-124 mimic or inhibitor resulted in a down-regulation and up-regulation of BCG-induced TLR signaling proteins and pro-inflammatory factors, respectively. Furthermore, we showed that miR-124 transcription was inversely correlated with MyD88 expression, which suggested a negative feedback mechanism of regulation between miR-124 and MyD88.

## Materials and Methods

### Cell culture

The A549 human alveolar type II epithelial cell line was purchased from the Type Culture Collection of the Chinese Academy of Sciences, Shanghai, China. The cells were cultured in F-12K (Gibco-Invitrogen, United States) nutrient mixture containing 10% (v/v) fetal bovine serum (FBS) (Hyclone, United States) supplemented with 0.1% penicillin/streptomycin at 37°C in 5% CO_2_ atmosphere.

### Synthesis of miRNA mimics and cell transfection

Based on the sequence of miR-124 in miRBase database (MIMAT0000422), miR-124 mimic (dsRNA oligonucleotides), negative control mimic (miR-124 nc) and miR-124 inhibitors (single- stranded chemically modified oligonucleotides) were synthesized in Ribobio Inc (Guangzhou, China). The oligonucleotides were transfected into A549 cells using Lipofectamine 2000 reagents per manufacturer's instructions (Invitrogen, United States). For cells cultured in a 6-well dish, 20 µM of miR-124 mimic, miR-124 nc or miR-124 inhibitor was transfected. The transfection mixture was removed and 2 ml/well of fresh medium was added at 6 h post-transfection, the transfected cells were cultured for an additional 24 h before they were harvested for analysis. Cells were transfected with pcDNA3.1 plasmid to serve as transfection controls for each experiment.

### Infection of A549 cells with BCG

*Mycobacterium bovis* BCG Beijing strain was purchased from the Center for Disease Control and Prevention (CCDC) of china (Beijing, China). The bacilli were grown at 37°C with shaking in Middle-brook 7H9 broth containing 10% albumin dextrose catalase supplement for 2 weeks, the bacterial cultures were then harvested by centrifugation at 500×g for 10 min and the cell pellets were resuspended in the BCG culture medium. The number of viable colony-forming units (CFU) of the culture was determined by plating serially diluted cultures on Middlebrook 7H11 plates supplemented with OADC enrichment (BD Biosciences Shanghai, Shanghai, China), and the bacterial colonies were counted after 4 weeks of culture [44]. Aliquots of the stock were stored at −80°C. A549 cells were infected with BCG at 6 h following transfection at a multiplicity of infection (MOI) of 3 and incubated at 37°C in a 5% CO_2_, humidified air atmosphere for 24 h before they were harvested for analysis.

### Infection of mice with BCG

Female C57/BL6 mice with six to eight weeks of age were purchased from the Animal facility of Ningxia Medical University (Yinchuan, China). The mice were housed in a special pathogen-free room and fed with food and water *ad libitum*. All experiments using animals were performed in accordance with the guidelines of the Chinese Council on Animal Care and approved by the Committee for Animal Care and Use of Ningxia University. Mice were randomly divided into two groups (8 mice per group). The mice of control group were intranasally instilled with 40 µl of PBS, and the animals of experimental group were instilled with 1×10^6^ colony-forming units (CFU) of BCG in 40 µl of PBS. 48 h after the infection, animals were euthanized and the lungs were harvested. The lung tissues were perfused with PBS to remove alveolar macrophages before they were homogenized for small RNA purification.

### Quantitative reverse transcription-PCR (qRT-PCR)

Small RNA was extracted from A549 cells or lung tissues using the RNAiso for small RNA kit, and total RNA was extracted using the AxyPrep Multisource Total RNA Miniprep kit per manufacturer's recommendations (Takara, Dalian, China). The RNA concentrations were determined using a NanoDrop instrument. The quality of RNA was assayed by calculation of the RNA integrity number (RIN) [45]. High quality RNA (RIN≥8.0) was used for reverse transcription of the first-strand cDNA synthesis by reverse transcription using M-MLV reverse transcriptase (TaKaRa, Dalian, China). The sequence of the primer used for reverse transcription of mature miR-124 included a stem-loop structure, which was designed based on the sequence of miR-124 (5′-CTCAACTGGTGTCGTGGAGTCGGCAATTCAGTTGAGGGCATTCA-3′). The qRT-PCR was performed using a Bio-Rad iQ5 lightcycler using a TaKaRa SYBR RT-PCR kit (Takara, Dalian, China). The RT-PCR primer set sequences used to detect the U6 promoter, β-actin, MyD88, NF-κB, IL-1α, IL-6, IL-8, IL-12α, TNF-α, IFN-β, and TRAF6 are listed in Table 1. The housekeeping gene, β-actin, was included to normalize for sample loading and RNA abundance. Relative expression was calculated as previously described using real-time PCR efficiencies and the crossing point deviation of an unknown sample *vs* a control [46]. The specificity of the primer sets was determined by sequencing the product of each qRT-PCR reaction.

**Table 1:** Primers used for qRT-PCR.

Primers	Sequence (5′-3′)	Tm	Size (bp)
β-actin	F: TTCGTGGATGCCACAGGACT	61°C	212
	R: GGGAAATCGTGCGTGACATT		
MyD88	F: CCAGTTTGTGCAGGAGATGA	61°C	288
	R: AGGATGCTGGGGAACTCTTT		
IL-1α	F: ATGTGACTGCCCAAGATGAAG	63°C	111
	R: CGTGAGTTTCCCAGAAGAAGA		
IL-6	F: GACAGCCACTCACCTCTTCAG	59°C	172
	R: CATCCATCTTTTTCAGCCATC		
IL-8	F: TTGCCAAGGAGTGCTAAAGAA	61°C	215
	R: GCCCTCTTCAAAAACTTCTCC		
IL-12α	F: CCTCTTTTATGATGGCCCTGT	63°C	88
	R: AGCTTTGCATTCATGGTCTTG		
TNF-α	F: TAGCC CATGTTGTAGCAAACC	63°C	136
	R: ATGAGGTACAGGCCCTCTGAT		
NF-κB	F: AGGAGAGGATGAAGGAGTTGTG	62°C	218
	R: CCAGAGTAGCCCAGTTTTGTC		
IFN-β	F: TTTGCTCTGGCACAACAGGTAGTA	63°C	131
	R: CCAAGCAAGTTGTAGCTCATGGAA		
TRAF6	F: TAGCCCTGGATTCTACACTGG	63°C	215
	R: CTTCGTGGTTTTGCCTTACAG		
U6	F: CTCGCTTCGGCAGCACA	59°C	94
	R: AACGCTTCACGAATTTGCGT		
miR-124	F: ACACTCCAGCTGGGTAAGGCACGCGGTGAATG	60°C	80
	R: CTCAACTGGTGTCGTGGA		

### 3′ untranslated region (3′UTR) luciferase reporters and miR-124 target validation

To validate the target transcripts of miR-124, reporter plasmids containing a coding gene luciferase upstream of the 3′UTR sequence of TLR6, MyD88, TRAF6 or TNF-α mRNA were generated by RiboBio Biotechnology (Guangzhou, China). The primers used for PCR amplification of the 3′UTR of each candidate transcript were designed based the sequence provided on the Genebank database and are listed in Table 2. Nested restriction sites were also included in the forward and reverse primers for subcloning. PCR-amplified 3′UTR fragments were cloned into the pMIR-Report vector (Invitrogen, Grand island, NY, USA) downstream of the luciferase reporter gene. In addition to constructs harboring the wild-type 3′UTR sequence, mutant variants (the miRs binding sequences were mutated, see detail in table 2) were also constructed. Luciferase reporter vectors, pMIR-Report/TLR6, pMIR-Report/MyD88, pMIR-Report/TRAF6 or pMIR-Report/TNF-α (harboring wild-type 3′UTR) and pMIR-Report/Mut-TLR6, pMIR-Report/Mut-MyD88, pMIR-Report/Mut-TRAF6 pMIR-Report/Mut-TNF-α (containing a mutated 3′UTR) were generated. The specificity of miR-124 to each 3′UTR target was tested by co-transfecting 293T cells with a miR-124 mimic and the reporter constructs. Luciferase activities were measured at 48 h after transfection by the relative activity of Renilla/firefly luciferase unit (RLU) using a Dual-Luciferase Reporter Assay (Promega, Madison, WI, USA). The experiments were performed independently in triplicate.

**Table 2:** Primers used for PCR amplification of 3′UTR fragments.

3′UTR	Wt or Mut	Sequence (5′→3′)	Fragment length	Genebank ID
TLR6	Wt	F: CTA GCGATCGC TTTGCTGGAGTCCGAGGGTGGGC	1185 bp	NM_006068
		R: GAATGCGGCCGCATCTGATTATTTAGAATCTGTGGC		
	Mut	F: CTA GCGATCGCTGTC***TCAGTA***ATTCAATAGTTAACTAC		
		R: GAATGCGGCCGCAAT***TACTGA***GACAGGCCAGCATGATC		
MyD88	Wt	F: CCGCTCGAGGTTCTGAGGCCCTGGGTGT	1740 bp	NM_002468.4
		R: GAATGCGGCCGCACACACAAGTTTCCAAGGTAGAATA		
	Mut	F: CCGCTCGAGTGAT***TCAGTAT***CAAAGTTATTTGTTTAC		
		R: GAATGCGGCCGCTG***ATACTGA***ATCAAGGTACAAAGTTGGT		
TRAF6	Wt	F: CTAGCGATCGCCACTTGCTCAAAAACAACTACC	1741bp	NM_004620.3
		R: GAATGCGGCCGCCAGGAACAGCAGCAGTGTAG		
	Mut	F: CTA GCGATCGC ACA ***CTCAGTA***TCCTTGCCCTGTTCTCA		
		R: GAATGCGGCCGC AGGA***TACTGAG***TGTTTTCTCCAGGTAG		
TNF-α	Wt	F: CCG CTCGAGGAACATCCAACCTTCCCAAAC	785 bp	NM_000594.3
		R: GAATGCGGCCGCTCTAAGCAAACTTTATTTCTCGC		
	Mut	F: CCG CTCGAGCTCT***CTCAGTA***CTTTTGATTATGTT		
		R: GAATGCGGCCGCAAG***TACTGAG*** AGAGGCCAGGG		

Wt: Wild-type; Mut: mutant; F: Forward primer; R: Reverse primer. The sequences of restriction sites were underlined. The sequences of mutated sites were bold and italic.

### Construction of vectors overexpressing MyD88 and inhibiting MyD88 expression

To generate a vector capable of overexpressing MyD88 in mammalian cells, human MyD88 cDNA was amplified by an RT-PCR strategy using total RNA from A549 cells according to the mRNA sequence of human MyD88 (NM_001172567). Restriction sites were introduced at each end of the PCR-amplified DNA fragment. The resultant of MyD88 fragment was cloned into the pcDNA3.1 backbone plasmid downstream of a CMV promoter (Invitrogen life technologies, United States).

To construct a vector for inhibiting MyD88 expression, an shRNA construct was generated to target the MyD88 cDNA sequence, 5′-GGAATGTGACTTCCAGACC-3′. Annealing the sense oligonucleotide, 5′-CACCGGAATGTGACTTCCAGACCTTCAAGAGAGGTCTGGAAGTCACATTCCTTTTTTG-3′, and the anti-sense oligonucleotide, 5′-GATCCAAAAAAGGAATGTGACTTCCAGACCTCTCTTGAAGGTCTGGAAGTCACATTCC-3′, resulted in a double stranded shRNA with Bbs I and BamH I sites at the 5′-end and 3′-end, respectively. The shRNA was then cloned into a pGPU6/GFP/Neo vector plasmid (GenePharma, Shanghai, China) to yield a pGPU6/GFP/Neo-MyD88/shRNA (referred to as MyD88 siRNA in this report).

### Immunoblotting analysis

Whole cell extracts were prepared by homogenizing cells in lysis buffer (50 mM Tris-HCl, pH 7.5, 5 mM EDTA, 150 mM NaCl, 0.5% NP-40) for 60 min on ice. The lysates were then centrifuged at 12,000 rpm for 10 min at 4°C, and the supernatants were collected for analysis. The soluble protein concentration was measured with Bio-Rad Protein Assay (Bio-Rad Laboratories, Richmond, CA) using bovine serum albumin (BSA) to generate a standard curve. The final clarified lysates (100 µg) were separated by 10% sodium dodecyl sulfate (SDS)-polyacrylamide gel (SDS-PAGE) and transferred to nitrocellulose membranes for Western blotting assay. The membrane was blocked in 5% fat-free dry milk in PBS containing 0.3% Tween-20, and probed with antibodies against GAPDH, TLR6, NFκB, TRAF6 and/or MyD88 (All antibodies were products of Santa Cruz Biotechnology, United States). The blots were then developed using the enhanced Western Bright ECL reagent (Advansta, United States).

### Enzyme-linked immunosorbent assay (ELISA)

To determine the IL-6 and TNF-α level in the medium of A549 cell cultures, the culture medium was collected after treatment and centrifuged at 1000×g for 5 min to pellet the cell debris. The supernatant was harvested and stored at −80°C prior to analysis. Human IL-6 and TNF-α levels in the supernatant were determined with appropriate ELISA kits per the manufacturer's instructions (Neobioscience Tech, Shenzheng, China). The protein concentration was first determined as pg/ml by generating a standard curve from protein provided in the kits. The data was presented as the fold(s) of change over the control groups.

### Bioinformatic analysis

The putative targets of miR-124 were predicted using the miRanda, PicTar and TargetScan target algorithms.

### Statistical Analysis

All data collected in this study was obtained from at least three independent experiments for each condition. SPSS15.0 analysis software and PRISM 5 were used for the statistic analysis. Statistical evaluation of the data was performed by one-way ANOVA when more than two groups were compared with a single control, and t-test for comparison of differences between the two groups. Significant differences were assigned to p values <0.05 and <0.01 denoted by * and **, respectively. Data was presented as the mean ± standard deviation (SD).

## Supporting Information

File S1 **Supporting table and figures.** **Table S1.** Potential targets of miR-124 in TLR signaling pathway predicted by bioinformatics tools. **Figure S1.** miR-124 regulates IFN-γ expression. The expression of IFN-γ was detected by qRT-PCR in A549 cells transfected with pcDNA3.1, miR-124 nc, miR-124 mimic or miR-124 inhibitor followed by infected without (A) or with BCG (B). Compared with pcDNA3.1 group *: p<0.05. Data represented the mean ± SD from three independent triplicated experiments (N = 6). **Figure S2.** miR-124 regulates IL-1α expression. The expression of IL-1α was detected by qRT-PCR in A549 cells transfected with pcDNA3.1, miR-124 nc, miR-124 mimic or miR-124 inhibitor followed by infected without (A) or with BCG (B). Compared with pcDNA3.1 group *: p<0.05. Data represented the mean ± SD from three independent triplicated experiments (N = 6). **Figure S3.** miR-124 regulates IL-8 expression. The expression of IL-8 was detected by qRT-PCR in A549 cells transfected with pcDNA3.1, miR-124 nc, miR-124 mimic or miR-124 inhibitor followed by infected without (A) or with BCG (B). Compared with pcDNA3.1 group *: p<0.05. Data represented the mean ± SD from three independent triplicated experiments (N = 6). **Figure S4.** miR-124 regulates IL-12α expression. The expression of IL-12α was detected by qRT-PCR in A549 cells transfected with pcDNA3.1, miR-124 nc, miR-124 mimic or miR-124 inhibitor followed by infected without (A) or with BCG (B). Compared with pcDNA3.1 group *: p<0.05. Data represented the mean ± SD from three independent triplicated experiments (N = 6).
(PDF)
