# Supplementary material for: Mycobacterium bovis BCG Triggered MyD88 Induces miR-124 Feedback Negatively Regulates Immune Response in Alveolar Epithelial Cells
Source: PLoS One. 2014 Apr 4;9(4):e92419. doi: 10.1371/journal.pone.0092419 (PMC3976256; doi:10.1371/journal.pone.0092419)

Supplemental figure legends

Figure S1. miR-124 regulates IFN-γ expression. The expression of IFN-γ was detected by qRT-PCR in A549 cells transfected with pcDNA3.1, miR-124 nc, miR-124 mimic or miR-124 inhibitor followed by infected without (A) or with BCG (B). Compared with pcDNA3.1 group *: $p<0.05$. Data represented the mean ± SD from three independent triplicated experiments (N=6).

Figure S2. miR-124 regulates IL-1α expression. The expression of IL-1α was detected by qRT-PCR in A549 cells transfected with pcDNA3.1, miR-124 nc, miR-124 mimic or miR-124 inhibitor followed by infected without (A) or with BCG (B). Compared with pcDNA3.1 group *: $p<0.05$. Data represented the mean ± SD from three independent triplicated experiments (N=6).

Figure S3. miR-124 regulates IL-8 expression. The expression of IL-8 was detected by qRT-PCR in A549 cells transfected with pcDNA3.1, miR-124 nc, miR-124 mimic or miR-124 inhibitor followed by infected without (A) or with BCG (B). Compared with pcDNA3.1 group *: $p<0.05$. Data represented the mean ± SD from three independent triplicated experiments (N=6).

Figure S4. miR-124 regulates IL-12α expression. The expression of IL-12α was detected by qRT-PCR in A549 cells transfected with pcDNA3.1, miR-124 nc, miR-124 mimic or miR-124 inhibitor followed by infected without (A) or with BCG (B). Compared with pcDNA3.1 group *: $p<0.05$. Data represented the mean ± SD from three independent triplicated experiments (N=6).

<table>
<tr><td></td><td>Supplemental table 1</td><td></td><td></td><td></td></tr>
<tr><td></td><td colspan="4">Predicted potential binding site (s) of miR-124 in the 3'-UTR of molecules of human TLR signaling pathway and interleukines</td></tr>
<tr><td>Signaling</td><td>Position of mRNA 3' UTR has-miR-124</td><td>predicted consequential pairing of target region (top) and miRNA (bottom)</td><td>seed match</td><td>probability of prenferential conservation</td></tr>
<tr><td>TLR1</td><td>none</td><td></td><td></td><td></td></tr>
<tr><td>TLR2</td><td>none</td><td></td><td></td><td></td></tr>
<tr><td>TLR3</td><td>none</td><td></td><td></td><td></td></tr>
<tr><td>TLR4</td><td>none</td><td></td><td></td><td></td></tr>
<tr><td>TLR5</td><td>none</td><td></td><td></td><td></td></tr>
<tr><td>TLR6</td><td>Position 1584-1590</td><td>5' ...UCAUGCUGGCCUGUCUGCCUUAU...</td><td></td><td>poorly conserved</td></tr>
<tr><td></td><td></td><td>||||||</td><td></td><td></td></tr>
<tr><td></td><td></td><td>3' CCGUAAGUGGCGCACGGAAU</td><td>7mer-1A</td><td></td></tr>
<tr><td>TLR7</td><td>none</td><td></td><td></td><td></td></tr>
<tr><td>TLR8</td><td>none</td><td></td><td></td><td></td></tr>
<tr><td>TLR9</td><td>none</td><td></td><td></td><td></td></tr>
<tr><td>TLR10</td><td>none</td><td></td><td></td><td></td></tr>
<tr><td>TLR11</td><td>unkown</td><td></td><td></td><td></td></tr>
<tr><td>TLR12</td><td>unkown</td><td></td><td></td><td></td></tr>
<tr><td>Myd88</td><td>Position 916-922</td><td>5' ...ACUUUGUACCUUGAUUGCCUUAC...</td><td></td><td>poorly conserved</td></tr>
<tr><td></td><td></td><td>||||||</td><td></td><td></td></tr>
<tr><td></td><td>poorly conserved</td><td>3' CCGUAAGUGGCGCACGGAAU</td><td>7mer-1A</td><td></td></tr>
<tr><td>TRAF6</td><td>Position 42-48 of TRAF6 3' UTR</td><td>5' ...UACCUGGAGAAAACAGUGCCUUU...</td><td></td><td></td></tr>
<tr><td></td><td></td><td>|||||||</td><td></td><td></td></tr>
<tr><td></td><td>conserved</td><td>3' CCGUAAGUGGCGCACGGAAU</td><td>7mer-m8</td><td></td></tr>
<tr><td></td><td>Position 1674-1680</td><td>5' ...UUGUGUCCCUCAGCU-GUGCCUUC...</td><td></td><td>poorly conserved</td></tr>
<tr><td></td><td></td><td>||| |||||||</td><td></td><td></td></tr>
<tr><td></td><td>poorly conserved</td><td>3' CCGUAAGUGGCGCACGGAAU</td><td>7mer-m8</td><td></td></tr>
<tr><td></td><td>Position 3849-3855</td><td>5' ...ACCUAAACAGUGCAAUGCCUUAA...</td><td></td><td></td></tr>
<tr><td></td><td></td><td>||||||</td><td></td><td></td></tr>
<tr><td></td><td>poorly conserved</td><td>3' CCGUAAGUGGCGCACGGAAU</td><td>7mer-1A</td><td></td></tr>
<tr><td>IRAK1</td><td>none</td><td></td><td></td><td></td></tr>
<tr><td>IRAK1BP1</td><td>none</td><td></td><td></td><td></td></tr>
<tr><td>IRAK2</td><td>none</td><td></td><td></td><td></td></tr>
<tr><td>IRAK3</td><td>Position 179-186</td><td>5' ...AAACCCUCAAACAGAGUGCCUUA...</td><td></td><td>poorly conserved</td></tr>
<tr><td></td><td></td><td>||| |||||||</td><td></td><td></td></tr>
<tr><td></td><td>poorly conserved</td><td>3' CCGUAAGUGGCG--CACGGAAU</td><td>8mer</td><td></td></tr>
<tr><td></td><td>Position 2779-2785</td><td>5' ...AUCCGUGAAAAUCGCUGCCUUAA...</td><td></td><td>poorly conserved</td></tr>
<tr><td></td><td></td><td>||||||</td><td></td><td></td></tr>
<tr><td></td><td>poorly conserved</td><td>3' CCGUAAGUGGCGCACGGAAU</td><td>7mer-1A</td><td></td></tr>
<tr><td>IRAK4</td><td>none</td><td></td><td></td><td></td></tr>
<tr><td>IL-1a</td><td>none</td><td></td><td></td><td></td></tr>
<tr><td>IL-1B</td><td>Position 134-140 of IL1B 3' UTR</td><td>5' ...CAAUGCCCAACUGCCUGCCUUAG...</td><td></td><td>poorly conserved</td></tr>
<tr><td></td><td></td><td>||||||</td><td></td><td></td></tr>
<tr><td></td><td>poorly conserved</td><td>3' CCGUAAGUGGCGCACGGAAU</td><td>7mer-1A</td><td></td></tr>
<tr><td>IL-2</td><td>none</td><td></td><td></td><td></td></tr>
<tr><td>IL-3</td><td>none</td><td></td><td></td><td></td></tr>
<tr><td>IL-4</td><td>none</td><td></td><td></td><td></td></tr>
<tr><td>IL-5</td><td>none</td><td></td><td></td><td></td></tr>
<tr><td>IL-6</td><td>none</td><td></td><td></td><td></td></tr>
<tr><td></td><td>none</td><td></td><td></td><td></td></tr>
<tr><td>IL-7</td><td>Position 605-611 of IL7 3' UTR</td><td>5' ...UGAAUGUGUAACACAGUGCCUUC...</td><td></td><td>poorly conserved</td></tr>
<tr><td></td><td></td><td>|||||||</td><td></td><td></td></tr>
<tr><td></td><td>poorly conserved</td><td>3' CCGUAAGUGGCGCACGGAAU</td><td>7mer-m8</td><td></td></tr>
<tr><td>IL-8</td><td>none</td><td></td><td></td><td></td></tr>
<tr><td>IL-9</td><td>none</td><td></td><td></td><td></td></tr>
<tr><td>IL-10</td><td>none</td><td></td><td></td><td></td></tr>
<tr><td>IL-11</td><td>Position 150-157</td><td>5' ...GCCUGGGGGGCAUCUGUGCCUUA...</td><td></td><td>poorly conserved</td></tr>
<tr><td></td><td></td><td>|||||||</td><td></td><td></td></tr>
<tr><td></td><td>conserved</td><td>3' CCGUAAGUGGCGCACGGAAU</td><td>8mer</td><td></td></tr>
<tr><td>IL-12a</td><td>none</td><td></td><td></td><td></td></tr>
<tr><td>IL-12b</td><td>none</td><td></td><td></td><td></td></tr>
<tr><td>IL-13</td><td>none</td><td></td><td></td><td></td></tr>
<tr><td>IL-15</td><td>none</td><td></td><td></td><td></td></tr>
<tr><td>IL-16</td><td>none</td><td></td><td></td><td></td></tr>
<tr><td>IL-17a,b,c,d,f</td><td>none</td><td></td><td></td><td></td></tr>
<tr><td>IL-18</td><td>none</td><td></td><td></td><td></td></tr>
<tr><td>IL-19</td><td>none</td><td></td><td></td><td></td></tr>
<tr><td>IL-20</td><td>none</td><td></td><td></td><td></td></tr>
<tr><td>IL-21</td><td>none</td><td></td><td></td><td></td></tr>
<tr><td>IL-23</td><td>none</td><td></td><td></td><td></td></tr>
<tr><td>IL-24</td><td>none</td><td></td><td></td><td></td></tr>
<tr><td>IL-25</td><td>none</td><td></td><td></td><td></td></tr>
<tr><td>IL-26</td><td>none</td><td></td><td></td><td></td></tr>
<tr><td>IL-27</td><td>none</td><td></td><td></td><td></td></tr>
<tr><td>IL-29</td><td>none</td><td></td><td></td><td></td></tr>
<tr><td>IL-31</td><td>none</td><td></td><td></td><td></td></tr>
<tr><td>IL-32</td><td>none</td><td></td><td></td><td></td></tr>
<tr><td>IL-33</td><td>none</td><td></td><td></td><td></td></tr>
<tr><td>IL-34</td><td>none</td><td></td><td></td><td></td></tr>
<tr><td>IL-36</td><td>none</td><td></td><td></td><td></td></tr>
<tr><td>IL-37</td><td>none</td><td></td><td></td><td></td></tr>
</table>

Ma, et al., 2013
Figure S1

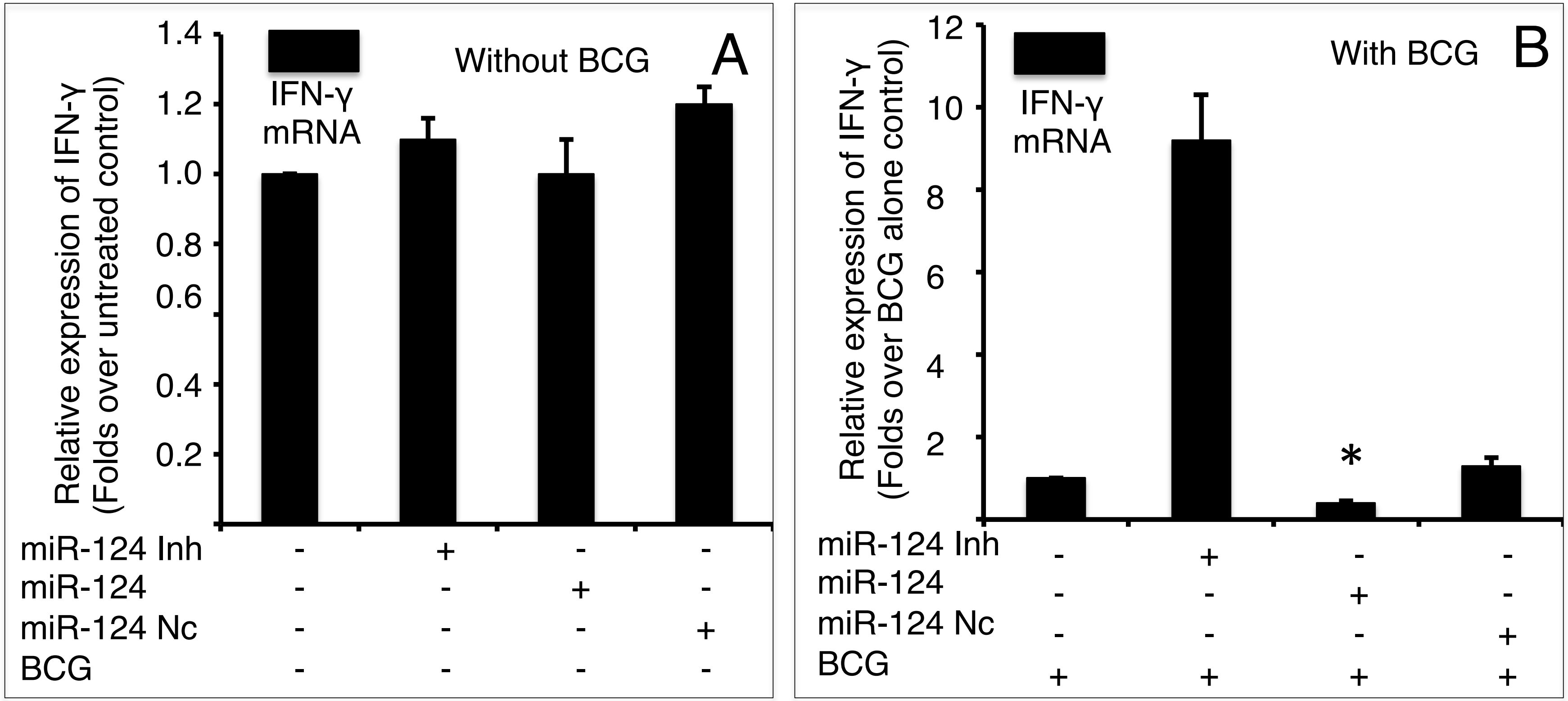

Ma, et al., 2013
Figure S2

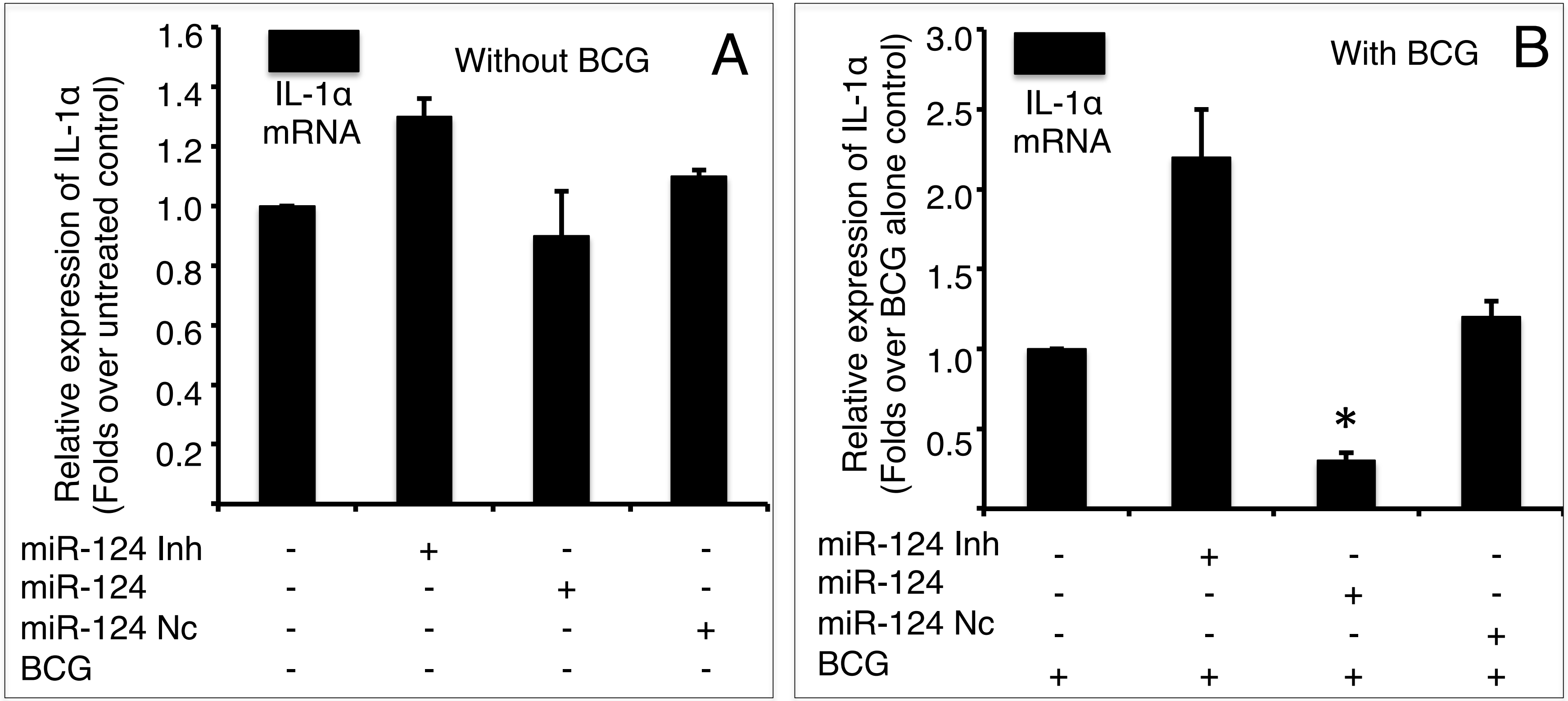

Ma, et al., 2013
Figure S3

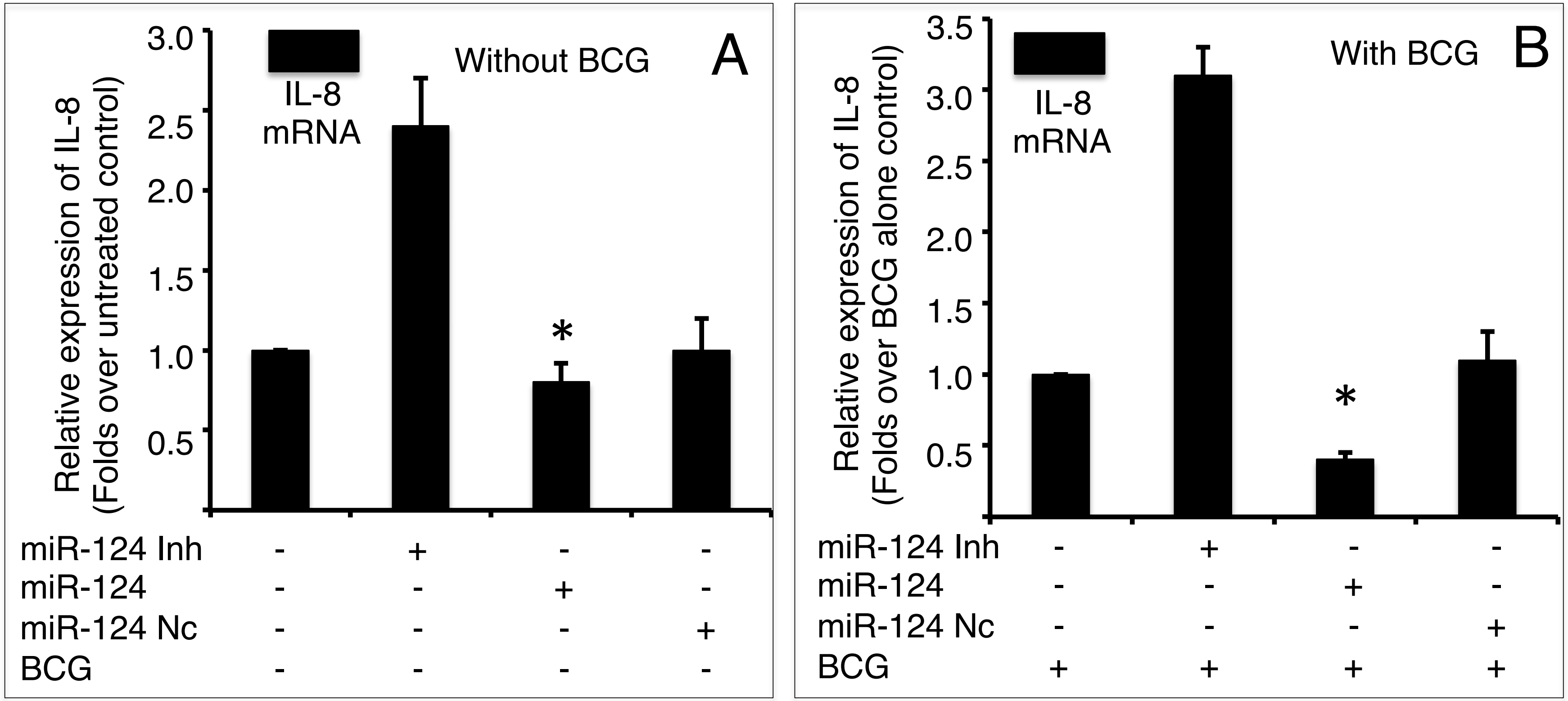

Ma, et al., 2013
Figure S4

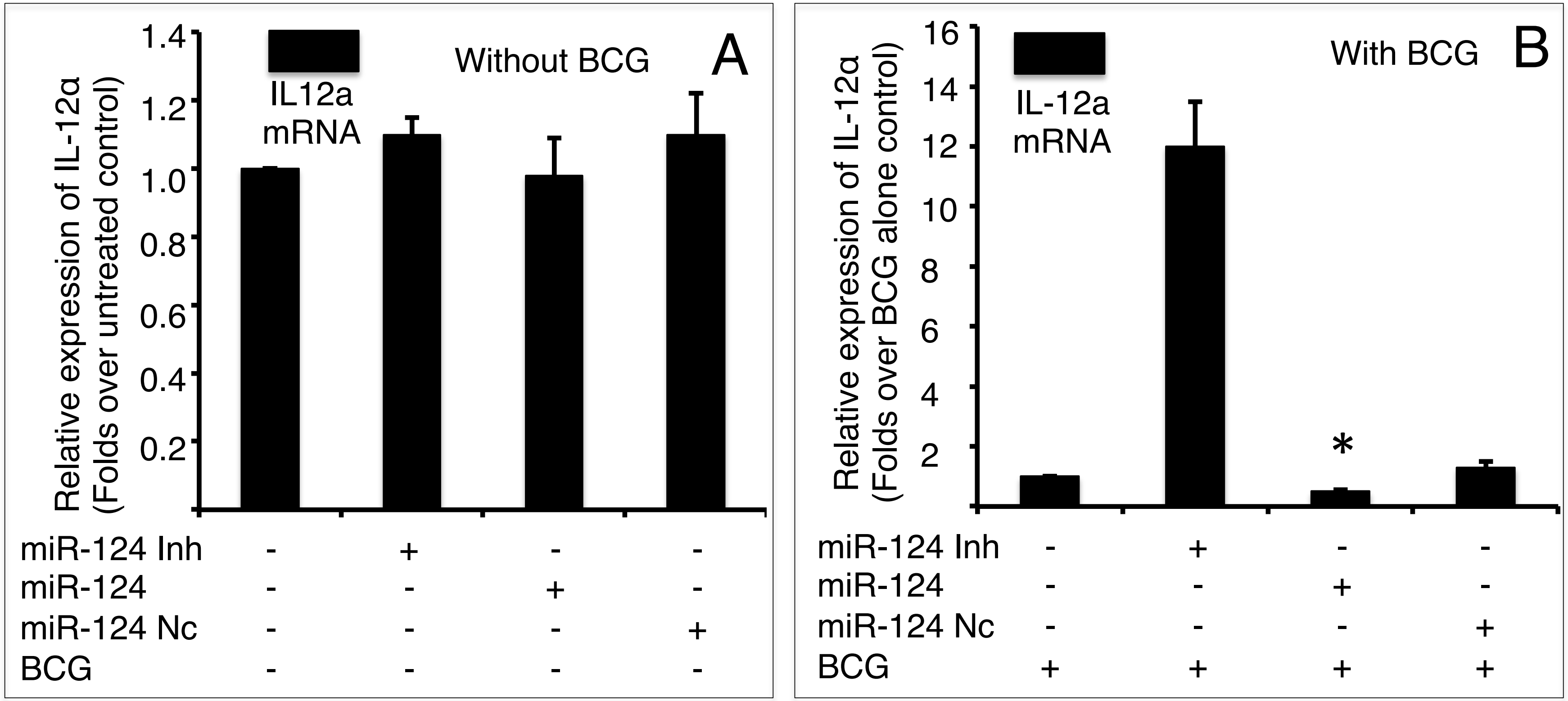

Supplement: File S1 — Supporting table and figures. Table S1. Potential targets of miR-124 in TLR signaling pathway predicted by bioinformatics tools. Figure S1. miR-124 regulates IFN-γ expression. The expression of IFN-γ was detected by qRT-PCR in A549 cells transfected with pcDNA3.1, miR-124 nc, miR-124 mimic or miR-124 inhibitor followed by infected without (A) or with BCG (B). Compared with pcDNA3.1 group *: p<0.05. Data represented the mean ± SD from three independent triplicated experiments (N = 6). Figure S2. miR-124 regulates IL-1α expression. The expression of IL-1α was detected by qRT-PCR in A549 cells transfected with pcDNA3.1, miR-124 nc, miR-124 mimic or miR-124 inhibitor followed by infected without (A) or with BCG (B). Compared with pcDNA3.1 group *: p<0.05. Data represented the mean ± SD from three independent triplicated experiments (N = 6). Figure S3. miR-124 regulates IL-8 expression. The expression of IL-8 was detected by qRT-PCR in A549 cells transfected with pcDNA3.1, miR-124 nc, miR-124 mimic or miR-124 inhibitor followed by infected without (A) or with BCG (B). Compared with pcDNA3.1 group *: p<0.05. Data represented the mean ± SD from three independent triplicated experiments (N = 6). Figure S4. miR-124 regulates IL-12α expression. The expression of IL-12α was detected by qRT-PCR in A549 cells transfected with pcDNA3.1, miR-124 nc, miR-124 mimic or miR-124 inhibitor followed by infected without (A) or with BCG (B). Compared with pcDNA3.1 group *: p<0.05. Data represented the mean ± SD from three independent triplicated experiments (N = 6). (PDF) [file pone.0092419.s001.pdf]
